# Recent advances in understanding chemotherapy-induced peripheral neuropathy

**DOI:** 10.12688/f1000research.21625.1

**Published:** 2020-03-11

**Authors:** Richard Gordon-Williams, Paul Farquhar-Smith

**Affiliations:** 1 Department of Pain Medicine, The Royal Marsden NHS Foundation Trust, Fulham Road, London, SW3 6JJ, UK

**Keywords:** Chemotherapy, Neuropathy, Pain, Survivorship

## Abstract

Chemotherapy-induced peripheral neuropathy (CIPN) is a common cause of pain and poor quality of life for those undergoing treatment for cancer and those surviving cancer. Many advances have been made in the pre-clinical science; despite this, these findings have not been translated into novel preventative measures and treatments for CIPN. This review aims to give an update on the pre-clinical science, preventative measures, assessment and treatment of CIPN.

## Introduction

The last decade has heralded improvements in cancer survival ^1^. However, persistent effects following the treatment of cancer can lead to pain and an impaired quality of life long after treatment has finished or cancer has been cured ^2^. Chemotherapy-induced peripheral neuropathy (CIPN) is one of those effects that can lead to a continuing symptom burden after treatment ^3^.

CIPN is characterised by the classic “glove and stocking” distribution of symptoms. After chemotherapy, 68% of patients have painful neuropathy at 6 months, improving to 33% at 1 year ^4^. Although different chemotherapies have variable characteristics, symptoms tend to be predominantly sensory. Sensory toxicity is the predominant feature as dorsal root ganglion (DRG), containing the sensory cell bodies, have a fenestrated endothelium that is more permeable than that found in the spinal cord, where the motor cell bodies lie. Sensory features are characterised by so-called “positive” and “negative” symptoms. “Negative” symptoms include numbness, loss of vibration sense, proprioception and deep tendon reflexes, whereas paraesthesia, dysaesthesia, and cold and mechanical hypersensitivity are referred to as “positive” symptoms.

The development of pain is also a common reason for dose reduction ^4, 5^, which may have implications for oncological outcome ^6^. The situation is further complicated by the effect of “coasting”, whereby the development of pain is delayed until after stopping the chemotherapy.

Despite advances in cancer treatment and survival, we still have much to learn about CIPN. It is important to recognise that CIPN is a heterogeneous population; it may be acute, such as the neuropathy commonly experienced with oxaliplatin, or chronic, lasting well beyond the end of treatment. Although there may be some overlap in features, it is likely that the underlying pathophysiology, clinical features and therefore its management differ substantially. Furthermore, not all CIPN is considered painful. This review will focus on the mechanisms but also deliberate on clinical features and treatment of chronic painful CIPN.

## Animal models

Animal models of CIPN have increased understanding of the pathophysiology of CIPN, yet a recent meta-analysis highlights problems with the current models and may help deliver more robust and valid models ^7^. For example, how do studies assessing short-term pain behaviours in animals without tumour burden model chronic CIPN? Pre-clinical studies often focus on the gain-of-function symptoms rather than the loss of function (for example, numbness) more common with chronic CIPN. Misrepresentation of the sexes is evident; 83% of animals used were male. Newer models have addressed some of these criticisms. Griffiths *et al*. describe a paclitaxel model of CIPN for 28 days with ethologically relevant behavioural tests that better mirror the clinical picture ^8^. Non-human primate models may be more similar to the human condition ^9^ but, owing to ethical and pragmatic issues, are not a feasible alternative to rodents.

## Mechanisms

The main classes of chemotherapeutics that cause neuropathy include the platinum-based anti-cancer therapies (oxaliplatin and cisplatin), vinca alkaloids (vincristine and vinblastine), taxanes (paclitaxel and docetaxel), proteasome inhibitors (bortezomib) and immunomodulatory drugs (thalidomide). These classes have differing anti-neoplastic mechanisms and likely different mechanisms for neuropathy. Evidence suggests that a number of mechanisms are shared between classes of chemotherapeutics, and most studies investigate the taxanes and the platinums. Currently, these mechanisms can be broadly separated into mitochondrial dysfunction and oxidative stress, microtubule disruption, neuroinflammation and immunological processes, and ion channel dysregulation.

### Mitochondrial and oxidative stress

Bioenergetic pathways, predominantly via the oxidation of glucose through the Krebs cycle within the mitochondria, are responsible for the generation of ATP. Chemotherapeutics commonly target nucleolar DNA and may also affect mitochondrial DNA. Indeed, targeting mitochondrial DNA as a principal therapy is an area of ongoing research ^10^. Whereas nucleolar DNA has well-established repair mechanisms, mitochondria do not. Flatters and Bennett showed that paclitaxel treatment in rats led to swollen vacuolated mitochondria that followed the course of pain-like behaviours for almost 3 months ^11^. Mitochondrial dysfunction within sensory neurones has also been demonstrated by other chemotherapeutics ^12– 15^. Krukowski *et al*. found that cisplatin-induced mechanical allodynia is associated with mitochondrial damage in DRG but that the loss of intra-epidermal nerve fibres (IENFs), seen in patients with CIPN, is related to bioenergetic deficits in peripheral nerves ^16^. Gregg *et al*. found that post-mortem platinum concentrations in patients who received platinum chemotherapy were highest in DRGs and demonstrated a linear relationship between DRG levels and cumulative dose ^17^, and levels were higher in patients with neuropathy. Animal data suggest a dose-dependent accumulation within the mitochondria of DRG neurones ^18^. Recently, gene expression analysis further supported mitochondrial dysfunction in patients who develop CIPN. Kober *et al*. ^19^ found that breast cancer patients who develop neuropathy after paclitaxel demonstrate differential expression in a number of pathways implicated in mitochondrial dysfunction, including oxidative stress ^20^. Additionally, genetic polymorphisms in anti-oxidant pathways have been associated with an increased incidence of CIPN ^21^.

Numerous animal studies indicate that chemotherapy worsens oxidative stress ^22, 23^. Furthermore, anti-oxidants prevent the development of mitochondrial dysfunction, IENF loss and pain-like behaviours in animal models ^24, 25^.

The anti-oxidant alpha-lipoic acid reduces neuropathy in patients with diabetes and also animal models of CIPN ^26^. Concurrent administration of alpha-lipoic acid reduces neuropathic symptoms secondary to bortezomib with less alteration to chemotherapy regimen secondary to adverse events ^27^. However, despite the neuroprotective effects of anti-oxidants *in vitro* studies ^28^, there is little clinical evidence for other nutraceutical anti-oxidants in the prevention of CIPN ^29^. Recently, however, a phase I trial showed that calmangafodipir, a manganese superoxide dismutase mimic that aids reactive oxygen species (ROS) degradation, reduces acute and chronic CIPN after oxaliplatin in patients ^30^ without affecting response to chemotherapy and life expectancy. Metformin can also reduce neuropathic behaviours via a reduction in oxidative stress ^13, 31, 32^. Metformin treatment in 40 patients receiving oxaliplatin reduced National Cancer Institute–Common Terminology Criteria for Adverse Events (NCI-CTCAE) grade 2 and 3 neuropathy with a moderate reduction in neurotoxicity score and a modest reduction in pain ^33^.

Mitochondria play a key role not only in ROS regulation but in numerous other cellular processes, including calcium buffering, apoptosis and energy production via oxidative phosphorylation. Duggett *et al*. have shown that whereas basal respiration and ATP turnover were unaffected in DRG mitochondria of paclitaxel treated rats, maximal respiration and spare reserve capacity were greatly reduced at peak pain behaviour ^12^. This indicates a reduced ability of these neurones to respond to stress, and the authors postulated that a switch to glycolysis could be an adaptive mechanism to reduce harmful ROS production.

Schwann cells play a crucial role in the regrowth of peripheral axons after injury; however, Nishida *et al*. found that accumulation of platinum compounds within Schwann cells was much lower than that in peripheral nerves and DRG ^18^. Conversely, Imai *et al*. suggested that *in vitro* platinum compounds cause mitochondrial dysfunction in Schwann cells at drug concentrations lower than those required to induce neurotoxicity ^34^, suggesting a greater role for mitochondrial dysfunction in Schwann cells in CIPN.

In animal models, treatment with pifithrin-μ, a molecule that suppresses mitochondrial damage, improves mitochondrial morphology, bioenergetics and IENF density while reducing pain behaviours ^14, 35^. Combined with evidence that it may act synergistically with the anti-cancer mechanisms of chemotherapeutics ^35, 36^, pifithrin-μ represents an exciting prospect in cancer care.

### Glia and neuroinflammation

Glia are key in maintaining homeostasis and immunity in the central nervous system in both health and disease. In models of non-chemotherapy-induced neuropathy, microglia have been found to play an integral role in the development of the pain state ^37, 38^. Oxaliplatin-treated rats displayed persistent mechanical allodynia, sensory deficits and decreased density of IENFs ^39^. Hu *et al*. showed a persistent activation of spinal cord microglia through strengthening of triggering receptor expressed on myeloid cells 2 (TREM2) signalling and demonstrated that either inhibiting microglia with minocycline or interrupting TREM2 signalling improved pain-like behaviours and IENF density ^40^. Furthermore, an agonist at the CB2 cannabinoid receptor, colocalised with spinal microglia, inhibited microgliosis and pain behaviours in an animal model of paclitaxel-induced neuropathy ^41^.

Despite these findings, astrogliosis rather than microgliosis is thought to be of greater importance to the development of CIPN ^42, 43^, while in some models, astrocyte inhibition with minocycline prevented the development of pain-like behaviours. But how would astrocyte activation lead to the development of CIPN? One proposed mechanism in a rat model of oxaliplatin-induced painful neuropathy is dysregulation of spinal adenosine kinase expression in astrocytes ^44^. This may lead to activation of NRLP3/interleukin 1 beta (NRLP3/IL1β) pathway, promoting dorsal horn neuronal excitability with concurrent suppression of the anti-inflammatory IL-10 system, leading to central sensitisation and pain behaviours ^44, 45^. Importantly, restoration of adenosine signalling with an A3AR adenosine receptor agonist prevents the development of both astrocytosis and pain behaviours ^45^. Another mechanism proposed in rodent models is through the alteration of sphingolipid signalling within astrocytes in the superficial layers of the dorsal horn of the spinal cord, an area concerned with nociceptive transmission ^46, 47^. Maladapted sphingolipid metabolism, through direct bortezomib effects and increased IL-1β, may increase glutamatergic transmission and consequently nociceptive transmission and pain behaviours ^47^.

Reasons for the discrepancies in the role glia play in CIPN remain unclear but the discrepancies may be due to variations in chemotherapy, species, time point and sex studied. Pain phenotype differs greatly between male and female patients, and the pathophysiology in animal models is also sex-dependent ^48^. In animal models of bortezomib-induced peripheral neuropathy, modulation of sphingolipid signalling attenuates pain behaviours in male but not female rodents ^47^. Additional examples of sexual dimorphism are found in paclitaxel-induced peripheral neuropathy, and Toll-like receptor 9 (TLR9) expression in macrophages infiltrating DRG plays a role in the development of pathophysiological changes and behaviours in male mice but not females ^49^. Macrophage infiltration into DRG and peripheral nerves has been seen in a number of animal models of CIPN, and as with other models of neuropathic pain, activation of TLR4 seems to be crucial ^50– 52^.

Clinically, minocycline treatment reduced only the acute pain syndrome associated with paclitaxel infusion but not the development of chronic CIPN ^53^. Additionally, in another phase 2 trial, minocycline failed to prevent oxaliplatin-induced peripheral neuropathy ^54^. Despite previous pre-clinical trials indicating minocycline’s efficacy at inhibiting astrocyte activation and pain behaviours, its actions have been ascribed predominantly to inhibition of microglia and not astrocytes ^55, 56^. Given the differential role that microglia may have in CIPN, minocycline’s lack of clinical efficacy may be of no surprise and neuroinflammation still represents a worthy area for continued research in the prevention of CIPN. Fingolimod, a drug used in the treatment of multiple sclerosis, downregulates the S1PR1 receptor found on astrocytes. Antagonism of this receptor has been shown to reverse immunochemical and behavioural changes in rodent models ^47^. This presents the exciting prospect of a potentially new mechanistic target with a readily available therapeutic agent; however, additional trials are required to assess both its effects on CIPN and importantly tumour activity.

### Ion channels

Pre-clinical studies have highlighted many chemotherapy-induced changes in ion channel expression, possibly driving behavioural changes in other neuropathic pain states ^57^.

Changes in sodium channel expression and their sensitisation increase spontaneous neuronal firing and decrease activation threshold ^58^, mechanisms possibly analogous to the allodynia, hyperalgesia and paroxysmal sensations of CIPN. In patients, sodium channel dysfunction is found in acute oxaliplatin toxicity ^59^, and sodium channel polymorphisms may have a causal role in the development of acute and possibly chronic CIPN ^60^. Furthermore, Na _v_1.7 channel has been found to be similarly upregulated in nociceptive neurones in both a rat model and patients with chronic paclitaxel-induced peripheral neuropathy ^61^. Although dysregulation of other sodium channels is seen in pre-clinical studies of CIPN ^62^, the clinical efficacy of sodium channel blockers has been disappointing ^63^.

Potassium channel dysregulation is present in animal models of CIPN ^64^. Acutely, oxaliplatin leads to the down-regulation of potassium channels in animal models ^62^, and Poupon *et al*. found that treatment with a riluzole (a potassium channel activator) prevents the development of persistent CIPN in mice ^65^. A phase 2 randomised controlled trial (RCT) investigating the efficacy of riluzole in the prevention of CIPN is under way ^66^. Transient receptor potential (TRP) channels are critical in temperature transduction. Oxaliplatin treatment leads to an increased expression of TRPA1, TRPV1 and TRPM8 in sensory neurones ^67^. Interestingly, suppression of TREK-1 and TRAAK potassium channels (and an increase in pro-excitatory Na _v_1.8 and HCN ion channels) is found on neurones expressing TRPM8, a receptor responsive to cold ^62^. This may present a mechanism through which menthol provides symptomatic relief and oxaliplatin produces cold hypersensitivity acutely.

Although calcium channel modulation has shown promise in animal models of CIPN ^68, 69^, no direct calcium channel blockers are in clinical use for neuropathic pain. Cisplatin causes an increase in the calcium channel alpha-2-delta subunit, the target of gabapentinoids ^70^, and both topical and systemic treatment with gabapentinoids have been found to be beneficial in rat models of CIPN ^71, 72^. Despite this, treatment with pregabalin for 3 days before and after each cycle of oxaliplatin failed to prevent CIPN in patients ^73^.

## Clinical features

### Risk factors

There are many potential predictors in the development of CIPN, including patient-related factors, such as increased age, pre-existing neuropathy, smoking status, and impaired renal function, and chemotherapy-related factors, such as type of chemotherapy, cumulative chemotherapy dose, concurrent chemotherapy treatment, and duration of infusion ^74– 76^. Certain cancers may cause a subclinical neuropathy which may predispose patients to CIPN and worsen outcomes ^77^.

Genetic markers have been implicated in chemotherapy-related toxicity, and a number of genome-wide association studies have looked at polymorphisms associated with CIPN. A number of polymorphisms have been identified, none of which (at present) has sufficient prognostic value to be of use in the clinical context ^78^. Argyriou *et al*. ^78^ called for improved methodology and more standardised diagnostic and severity grading to better inform future studies.

### Assessment of CIPN

Despite challenges in prevention and treatment, assessment for CIPN should occur before, during and after chemotherapy. Assessment should include (1) diagnosis (including possible differential diagnoses), (2) severity (including functional impairment) and (3) time course of symptoms and relationship to chemotherapy.

Diagnosis of CIPN requires a full history and examination ( Table 1). Within the history, it is important to determine pre-existing risk factors for neuropathy such as diabetes, vitamin deficiency, alcohol use and previous chemotherapy. Blood tests, including a full blood count, comprehensive metabolic profile, measurement of erythrocyte sedimentation rate, fasting blood glucose, vitamin B _12_, and thyroid-stimulating hormone levels, should be considered to help rule out other causative or contributory causes for neuropathy.

**Table 1.** Key elements in history and examination.

History	Examination
• Details of chemotherapy regimen • Number of cycles, dose and cumulative dose • Onset of symptoms in relation to chemotherapy • “Coasting” assessment (neuropathy occurring or worsening after chemotherapy cessation) • Evidence of change over time (better or worse) Symptoms • Distribution (hands, feet or more proximal) • Numbness, paraesthesia, pain, spontaneous or evoked • Motor or sympathetic dysfunction • Functionality and interference on activities	Sensation • Light touch • Pinprick or painful stimulus • Vibration sense • Cold/hot sensation Other • Deep tendon reflexes • Motor power • Balance

Painful CIPN is a subset that may benefit from further characterisation with multidimensional pain assessment tools. The McGill Pain Questionnaire (MPQ) and the Brief Pain Inventory (BPI) have been validated for use in cancer pain ^79^ and although they both assess sensory aspects, including severity, the BPI also assesses impact on function. Likewise, screening tools may aid in the assessment of neuropathic pain. Two such tools, the Leeds Assessment of Neuropathic Symptoms and Signs (LANSS) and Douleur Neuropathique 4 (DN4) ^80, 81^, have good sensitivity and specificity in cancer pain but are not validated in CIPN. Furthermore, although chemotherapy is a common treatment for childhood cancers and the subsequent neuropathy may differ in phenotype from that of adults ^82, 83^, there are few validated tools for the assessment of CIPN in children. One such score is the paediatric-modified total neuropathy score (ped-mTNS), which has been validated in a small group of children undergoing vincristine or cisplatin chemotherapy for leukaemia ^84^; however, the 2008 Pediatric Initiative on Methods, Measurement, and Pain Assessment in Clinical Trials (PedIMMPACT) called for the development of reliable and valid tools for use in children ^85^.

Numerous tools have been developed for the assessment of CIPN; however, there is notable inter-observer variation between these scales. Clinical rated scales such as the Ajani scale, World Health Organization, Eastern Cooperative Oncology Group neuropathy scale, and NCI-Common Toxicity Criteria (NCI-CTC) have limited assessment of pain ^86^ and may not truly reflect the incidence of adverse neuropathy, leading to inappropriate treatment reduction or cessation. Furthermore, clinician-rated neuropathy scales underestimate the severity of CIPN when compared with patient-reported measures ^87^. In a recent systematic review, Haryani *et al*. suggested that, owing to their psychometric properties and practicality, the Functional Assessment of Cancer Therapy/Gynecologic Oncology Group-Neurotoxicity (Fact/GOG-Ntx) and total neuropathy score (TNS) (see below) were the most appropriate assessment tools available ^88^. Yet other studies are contradictory; this is in line with a recent DELPHI survey which showed there is little consensus amongst clinicians ^89^.

Fact/GOG-Ntx was developed for assessing the impact of neuropathy on quality of life after chemotherapy for gynaecological cancer and consists of questions on physical, social, emotional and functional wellbeing with an additional 11-question neurotoxicity subscale. This subscale has been used independently and demonstrates good sensitivity in diagnosis and responsiveness to treatment ^90^ and has been validated in other non-gynaecological, non-cisplatin/paclitaxel patients. Further shortening the neurotoxic subscale to four sensory questions maintains the validity and sensitivity while reducing the burden of patient questions ^91^.

The Patient Neurotoxicity Questionnaire (PNQ) evaluates sensory, motor and functional components of neuropathy with good sensitivity to change over time and showed improved reporting of CIPN when compared with clinician reporting tools such as the NCI-CTC (see above). Importantly, the PNQ assesses the impact of neuropathy on 22 activities (such as fastening buttons or typing) that are not assessed with other tools, thus representing a more holistic patient-centred assessment of neuropathy.

The TNS is an eight-item score of patient report of neuropathic symptoms, examination findings to pinprick, vibration and deep tendon reflexes and nerve conduction studies (NCSs). A shortened version without the electrophysiological factors has been validated: the TNSc (clinical version of the TNS) ^92^; both have been shown to be more sensitive to CIPN changes than NCI-CTC and comparable in changes to quality-of-life measures ^93^. Importantly, TNS delivers both clinician- and patient-rated components. Quality-of-life measures are commonly not assessed in many CIPN tools. The European Organization of Research and Treatment of Cancer (EORTC), 20-item quality-of-life questionnaire, is sensitive to changes in quality of life secondary to CIPN ^94^.

### Investigations

There has been a great deal of interest in phenotyping CIPN by using minimally invasive tools such as NCSs, quantitative sensory testing (QST) and IENF density. It seems sensible that underlying mechanisms may translate to differing patterns of neuronal loss and therefore differences in functional deficits, yet in practice this theory is not robust. Traditionally, CIPN has been characterised as a predominant sensory neuropathy effecting large myelinated fibre function, and nerve biopsies from patients with cisplatin- and paclitaxel-induced neuropathy show a loss of large fibres with axonal atrophy and secondary demyelination ^95, 96^.

Platinum chemotherapeutics cause neuronal cycle arrest within the DRG and therefore likely cause a neuronopathy (also referred to as ganglionopathy) and anterograde neuronal degeneration. On NCS, this would manifest as non-length-dependent neuropathy affecting both the proximal and distal neurone. In contrast, chemotherapeutics interfering with mitochondrial or microtubule function impair axonal transportation giving a length-dependent axonal polyneuropathy, leading to a die back of intraepidermal nerve fibres. However, owing to poor correlation with clinical symptoms, NCSs cannot be routinely recommended. Furthermore, NCSs assess predominantly large-fibre function, missing small-fibre changes that may occur with painful CIPN.

Owing to its ability to assess large- and small-fibre types, QST may be of use in the phenotyping of neuropathic pain ^97^ and therefore has been proposed as a useful tool in CIPN ^98^. In patients with paclitaxel-induced peripheral neuropathy, the reduction in light touch and vibration detection thresholds seen in hands and feet supports the mechanism of paclitaxel causing a distal neuropathy predominantly effecting the large, non-nociceptive neurones ^99^. Additionally, some report that thermal detection thresholds and pinprick detection are minimally affected, indicating that small-fibre function is preserved in this group. This is in contrast to findings in patients with vincristine and bortezomib-induced neuropathy, with some studies reporting changes in pinprick perception and warm detection thresholds suggesting small, nociceptive fibre dysfunction in this group of patients ^100, 101^.

Pre-existing QST sensory deficits increase the risk of developing CIPN ^102^; in some cases, cancer itself may be responsible for QST changes ^103^. Although the QST sensory profile may differ between agents ^104, 105^, QST profiles for painful and painless CIPN may be similar ^106^ and changes in QST may occur later than symptoms develop ^107^. Furthermore, QST requires expertise and time and consequently is not commonly used in routine clinical practice for the evaluation of CIPN.

Skin punch biopsy can inform the diagnosis of small-fibre neuropathies. In CIPN, similar to other small-fibre neuropathies, IENF loss is observed ^108^. Taking comparative distal thigh and distal leg punch biopsies can help differentiate between a length-dependent neuropathy or a neuronopathy; however, evaluating CIPN using IENF densities has been found to be unreliable; there is a large overlap between different chemotherapeutics, and results conflict with other assessment tools ^109– 111^. Furthermore, although punch biopsy can be repeated, it is time-consuming and invasive and IENF density has been found to be a poor correlate of pain ^112^.

Other techniques for assessing neuropathy have yet to be fully validated. Nevertheless, simple bedside measures such as vibration sense, light touch and pinprick have good validity in the measurement of neuropathy ^113^.

## Prevention

Reducing regional perfusion (cryotherapy) may reduce CIPN; cooling gloves and stockings have been shown to reduce the risk of desquamation and nail changes associated with chemotherapy. Of the three published trials, only one showed benefit ^114^. Owing to poorly tolerated treatment or a greater-than-expected control group response, the other studies were negative ^115– 117^.

In 2014, the American Society of Clinical Oncology evaluated 42 studies while developing guidelines on the prevention of CIPN ^63^. Owing to a lack of high-quality data, they were unable to make any recommendations and encouraged additional research.

## Treatment

### Pharmacological

RCT evidence of treatments in CIPN suggested that duloxetine is the only anti-neuropathic agent with evidence of benefit ^118^. Many CIPN RCTs fail to meet the IMMPACT guidelines for outcome measures in clinical trials ^119, 120^. Nevertheless, a recent comparative study showed that venlafaxine and duloxetine reduced pain in established CIPN ^121^. Careful phenotyping may help, as demonstrated with the improved efficacy of oxcarbazepine in the “irritable nociceptor” subgroup ^122^. Phenotyping patients for biological and psychosocial characteristics may give additional insight ^122– 125^.

Topical treatments are an attractive option for the management of CIPN. A small non-randomised study of topical menthol in 52 patients showed improved BPI scores ^126^, and combination therapy with baclofen, amitriptyline and ketamine showed an improvement on some of the EORTC QLQ-CIPN-20 measures ^127^. Topical 8% capsaicin patch application following CIPN has been shown to improve continuous pain, neuropathic pain symptoms, and patient global impression of change ^128^. This treatment has also been found to improve IENF density, suggesting underlying disease modification ^128^.

There is increasing enthusiasm for the use of cannabinoids in the treatment of many chronic pain states. Agonism at CB1 and CB2 receptors has shown analgesia in rodent models of CIPN ^129– 133^ but these findings have not translated into evidence of clinical efficacy. One published pilot study of nabiximols (THC:CBD mix) in 15 patients with CIPN ^134^ showed no significant improvement in pain, but a 2-point decrease over placebo was seen in five patients classified as “responders” ^134^.

Without specific evidence for CIPN, clinicians extrapolate treatments from other neuropathic pain states ^135^. Interestingly, strong opioids have some of the best “numbers needed to treat” (NNTs) for neuropathic pain (NNT 4.3, 95% confidence interval 3.4–5.8) ^135^. Some clinicians may advocate the use of opioids in CIPN, however with increasing survivorship amongst patients with cancer, the possible benefits of opioids should be continually weighed up against the risk of long-term opioid therapy ^2^.

### Non-pharmacological

Neuromodulation has shown promise in various neuropathic pain states ^136^. A number of case reports indicate that neuromodulation may help refractory CIPN ^137, 138^, but RCT data are lacking.

A recent study found that the use of wireless transcutaneous electrical nerve stimulation significantly improved some measures of CIPN, including pain, numbness and tingling ^139^. Furthermore, scrambler therapy (a novel transcutaneous neurostimulation technique) has been postulated as a potential treatment ^140^ but was no more effective than sham therapy in a recent RCT ^141^.

### Acupuncture

A Cochrane Review of the efficacy of acupuncture in the treatment of cancer pain showed insufficient evidence of its efficacy ^142^. Since then, a number of trials of acupuncture in CIPN have demonstrated improvements in several domains ^143– 145^. A systematic review concluded that there was insufficient evidence to recommend acupuncture for the treatment of CIPN ^146^, although low risk of harm and possible benefit may allow its pragmatic use in painful CIPN.

### Physical therapy

Exercise has been shown to improve a number of facets that contribute to morbidity associated with CIPN, including balance and strength ^147, 148^, numbness, tingling, and hot and cold sensations ^149^. One study found that, on analysis of the quality-of-life data, exercise had a moderate effect on pain in patients undergoing chemotherapy; however, this was not limited to CIPN ^150^.

### Psychological therapy

Psychological factors have been shown to play a role in both the initiation and maintenance of a number of chronic pain states ^151^. The activity of duloxetine, via enhancing descending inhibitory pathways, suggests that alteration of mood may play a role. In favour of this viewpoint, a study of 111 patients who received treatment for breast cancer found that pre-existing anxiety and pre-therapy numbness were the only factors to predict CIPN eight months later ^152^. Knoerl *et al*. found that an eight-week web-based cognitive behavioural programme led to modest improvements in worst pain with no differences in mean pain ^153^. It was hypothesised that this would be due to improvements in fatigue, anxiety, sleep-related factors, or depression; however, a follow-up analysis was unable to substantiate these findings ^154^.

## Future directions

Pre-clinical studies have shown that antagonism of the sigma 1 receptor (present on mitochondrial endoplasmic reticulum) is able to reduce mitochondrial structural changes and pain behaviours that occur in CIPN. A phase II clinical trial found that sigma 1 antagonist treatment during FOLFOX chemotherapy diminished cold hypersensitivity, reduced the dropout rate and allowed a higher cumulative dose of oxaliplatin ^155^. Although the long-term pain outcomes are not known, this highlights a pathway for potential therapeutics that could improve CIPN.

## Summary

Despite an ever-expanding body of literature behind the pathophysiology and treatment of CIPN, new treatment options are still limited, and a proportion of patients continue to have difficulty controlling symptoms causing a significant impact on quality of life. Guided by the pre-clinical literature, novel targets that may help prevent CIPN are beginning to emerge. However, with continual advancements in chemotherapeutic agents with novel mechanisms, it is important that ongoing development of treatments for CIPN continue.
